# Supplementary material for: A generalized class of estimators for sensitive variable in the presence of measurement error and non-response
Source: PLoS One. 2022 Jan 19;17(1):e0261561. doi: 10.1371/journal.pone.0261561 (PMC8769360; doi:10.1371/journal.pone.0261561)
Supplement: Attachment — Submitted filename: Response to reviewer plos.docx [file pone.0261561.s002.docx]

Response to the reviewer:

Please use the space provided to explain your answers to the questions above. You may also include additional comments for the author, including concerns about dual publication, research ethics, or publication ethics. (Please upload your review as an attachment if it exceeds 20,000 characters)

Reviewer #1: In this papers, the authors studied a general class of estimators is proposed for the finite population mean of a sensitive variable. The paper has some weak points. The authors used some techniques based on classic estimators, without cite news trends in this fields (e.g., fractal-wavelet models). Thus I suggest to add (at least) the references below.

Suggested references are not relevant to the present paper.

Moreover, the English needs several improvements. I suggest a native English speaker. In particular, Abstract and Introduction cannot be publish in the current version. Finally, formulas must be rewritten in a more concise style.

Done
